# High-fidelity replication of thermoplastic microneedles with open microfluidic channels

**DOI:** 10.1038/micronano.2017.34

**Published:** 2017-10-09

**Authors:** Zahra Faraji Rad, Robert E. Nordon, Carl J. Anthony, Lynne Bilston, Philip D. Prewett, Ji-Youn Arns, Christoph H. Arns, Liangchi Zhang, Graham J. Davies

**Affiliations:** 1 Graduate School of Biomedical Engineering, University of New South Wales, Sydney, NSW 2052, Australia; 2 School of Mechanical Engineering, University of Birmingham, Birmingham B15 2TT, UK; 3 School of Mechanical and Manufacturing Engineering, University of New South Wales, Sydney, NSW 2052, Australia; 4 Prince of Wales Clinical School, University of New South Wales, Sydney, NSW 2052, Australia; 5 School of Petroleum Engineering, University of New South Wales, Sydney, NSW 2052, Australia

**Keywords:** drug delivery, laser lithography, microneedles, point-of-care diagnostics, soft embossing

## Abstract

Development of microneedles for unskilled and painless collection of blood or drug delivery addresses the quality of healthcare through early intervention at point-of-care. Microneedles with submicron to millimeter features have been fabricated from materials such as metals, silicon, and polymers by subtractive machining or etching. However, to date, large-scale manufacture of hollow microneedles has been limited by the cost and complexity of microfabrication techniques. This paper reports a novel manufacturing method that may overcome the complexity of hollow microneedle fabrication. Prototype microneedles with open microfluidic channels are fabricated by laser stereolithography. Thermoplastic replicas are manufactured from these templates by soft-embossing with high fidelity at submicron resolution. The manufacturing advantages are (a) direct printing from computer-aided design (CAD) drawing without the constraints imposed by subtractive machining or etching processes, (b) high-fidelity replication of prototype geometries with multiple reuses of elastomeric molds, (c) shorter manufacturing time compared to three-dimensional stereolithography, and (d) integration of microneedles with open-channel microfluidics. Future work will address development of open-channel microfluidics for drug delivery, fluid sampling and analysis.

## Introduction

Microneedle devices offer an alternative to the hypodermic needle for blood extraction and injection of drugs. Microneedles are designed to penetrate skin and capillaries without causing pain or the need for medical expertise, so that diagnosis and treatment can be administered at point-of-care^1^. Undoubtedly, the most challenging problem for this field is development of low cost manufacturing methods that will lead to clinical translation of microneedle technology. The manufacturing processes commonly utilized for microneedles fabrication are injection molding^2^, reactive ion etching^3^, chemical wet etching^4^, micromolding^5^, and two-photon polymerization^6^. The choice of fabrication method depends on the manufacturing material, access to manufacturing technology, and the intended application (drug delivery or fluid sampling). Polymeric materials are receiving some interest from the medical industry because of their ease of manufacture, low cost and favorable biological and mechanical properties^2,7^. Hollow bore microneedles are designed to deliver or collect fluid across the skin. The fluid may be a drug, vaccine, blood, or interstitial fluid (ISF). Solid microneedles may only be used for drug or vaccine delivery. Solid microneedles are simpler to manufacture than hollow microneedles^8^, with elution of drug into the tissues after skin penetration. open-channel microneedles draw fluid by capillary tension and are simpler to manufacture compared to internal bore designs which are difficult to replicate by molding. Open-channel microneedles provide two-dimensional flows of fluids, which can be used for both extracting biological fluid and delivering drugs. Single *in-plane* open-channel microneedles with 2D features have been manufactured by numerically controlled milling and lithography^9,10^.

Interstitial fluid can be extracted from 50 μm beneath the skin surface^11^, while blood collection requires penetration to a depth of at least 400 μm to gain access to subcutaneous capillaries^12,13^. Microneedle lengths for painless blood collection are usually 400–900 μm^12,13^, though microneedles >1 mm have also been reported^9,14^. Skin indentation, dermatoglyphics (small wrinkles) and hair on the surface of skin limits the depth of penetration^15^. Compaction of the skin layer when pressure is applied may limit the hydraulic conductivity of hollow microneedles^15^. An understanding of the biomechanics of skin insertion is important for specification of microneedle tip sharpness, mechanical stability and actuators for microneedle insertion.

The manufacture of long microneedles by subtractive manufacturing methods is technically challenging^3,4,9,10^, so fabrication methods have dictated microneedle geometry rather than biomechanical and physical design considerations. However, we present a simple and versatile fabrication process directly linking three-dimensional (3D) modeling and simulation with microscale printing and replication. The process we have initiated involves microstructures fabricated by 3D stereolithography directly from CAD drawings, which were then replicated by soft embossing. Micro-computed tomography (Micro-CT) demonstrated that feature sizes were within 4% of the CAD drawing specification. The mechanical stability of microneedles was determined by finite element analysis (FEA) and physical compression tests. These data were used to predict if microneedles are likely to fail during skin penetration. Surface energy and channel dimensions determine the rate of filling of microneedle open channels and reservoirs with aqueous solutions. A 4×4 array of open-channel microneedles connected to individual two nanolitre reservoirs was tested *in vivo*. Multiphoton fluorescence microscopy was used to demonstrate delivery of fluorescein tracer into the skin of excised rabbit ears. These initial proof-of-concept studies demonstrate the potential application of 3D microneedle prototyping and embossing for drug delivery and point-of-care diagnostics.

## Materials and methods

### Open-channel microneedle design

Microneedles presented in this paper comprise a cylindrical body, an ultra-sharp pointed tip to penetrate tissue, an open channel extending along a side of the body from the tip to the base of the microneedle, and a flange-shaped base connecting the needle to the back plane, with an open channel connecting the shaft open channel to a through hole and/or reservoir. The design was based on solid and fluid mechanics considerations. Fluid flow channels had to be partially open so that the microneedle array can demold from an elastomeric negative mold.

The geometric details of these designs are based upon an understanding of the material properties of the material and fluid dynamic properties of open-channel flow. The microneedles are 700 μm in height and the internal diameter of microneedles open channels are 30 μm. This diameter needs to be large enough to ensure the flow of cells especially larger cells such as leukocytes (white blood cells). Other parameters that determine flow in a microchannel include blood viscosity, hydrodynamic diameter, contact angle, and driving forces such as surface tension.

### Fabrication of master microneedles

Polymeric master microneedles with the geometric designs presented in this paper were fabricated by 3D laser lithography using the Photonic Professional GT system (Nanoscribe GmbH, Karlsruhe, Germany). The direct laser writing (DLW) technique also known as two-photon polymerization (TPP) or 3D laser lithography is a nonlinear optical process based on two-photon absorption (TPA) theory. The Nanoscribe system is equipped with a pulsed erbium-doped femtosecond (frequency-doubled) fiber laser source with a center wavelength of 780 nm for the exposure of the photoresist. At the pulse length of 100–200 femtosecond the laser power ranges between 50–150 mW (Ref. 16). For fabrication of several types of microneedles CAD models were generated by SolidWorks software (Dassault Systems SolidWorks Corporation, Concord, NH, USA) in stereolithography (STL) file format and imported to the software package Describe (Nanoscribe GmbH, Germany) for scripting of writing parameters. The laser beam was focused into the negative-tone photoresist, IP-S (Nanoscribe GmbH, Karlsruhe, Germany), using a Dip-in laser lithography (DiLL) objective with ×25 magnifications and NA=0.8.

In this process, the objective lens is directly dipped into the liquid and uncured photoresist acts as both photosensitive and immersion medium in an inverted fabrication manner. The refractive index of the photoresist defines the focal intensity distribution. For the DiLL process the objective working distance does not limit the height of the sample; therefore, structures with millimeters heights can be fabricated. A drop of resist was cast on the silicon substrate; IP-S exhibited good adhesion on the silicon substrate, and loaded onto the system. Microneedle arrays were written in galvo scan mode (*XY*) and piezo *Z* offsetting mode. The arrays were split into blocks of 317 μm×312 μm×20 μm (*XYZ*) within the working range of the galvo scan mode and stitched together. The Laser power was 100 mW, scan speed 6 cm s^−1^, minimum and maximum slicing distance 0.3 and 0.5 μm, respectively, were chosen after process optimization. After exposure, the structures were developed in propylene glycol monomethyl ether acetate (PGMEA) bath for 30 min plus a 3 min isopropyl alcohol (IPA) rinse followed by 20 min flood exposure through a UV light source with 16 mW cm^−2^ intensity to further crosslink the photosensitive material (See Supplementary Discussion for process optimization).

### Casting of negative elastomeric mold

A ‘soft’ negative impression of the masters was cast using silicone elastomer polydimethylsiloxane (PDMS) (SYLGARD 184 Silicone Elastomer Kit, Dow Corning, Midland, MI, USA) with a base/curing agent ratio of 10:1 in a Petri dish. The mixture was degassed in a vacuum chamber for 60 min to suppress formation of air bubbles during the subsequent curing stage in a standard laboratory oven at 60 °C overnight. The cured PDMS molds were peeled off the master prototypes to be used as negative molds for microneedles replication.

### Embossing thermoplastic materials using negative elastomeric molds

Thermoplastic microneedle replicas were created by a soft embossing process, which was performed on a rheometer (Kinexus Rheometer, Malvern Instruments Ltd., Worcestershire, UK) using the PDMS-negative molds. ‘Soft’ negative impressions of the master prototype microneedles were cast using the silicone elastomer, PDMS. One or two thermoplastic pellets (cyclo-olefin polymer, Zeonor 1060R) were loaded onto each cavity of PDMS-negative molds and placed between two 20 mm diameter stainless steel plates. The upper plate was lowered until the plates were in contact and heated up to 160 °C, 60 °C above the glass transition temperature of the thermoplastic (*T*_g_=100 °C). This molding temperature decreases the viscosity of the molten thermoplastic so that it easily penetrates the negative mold cavities. The upper plate was then lowered further as the thermoplastic melted, until a specified target force was reached. On average, a maximum force of 19.52±0.64 N (mean±standard deviation) was applied during this embossing process. In order to achieve consistent and uniform embossing, the molding temperature was fixed at 160 °C for around 15 min throughout the embossing process^17^, while the desired gap between the plates was achieved by applying a calibrated force.

Then the mold and molten polymer were cooled down to 10–15 °C which was maintained for 10–15 min with constant force (1.6 N) before demolding. Solidified thermoplastic microneedle arrays were separated from the PDMS elastomeric mold without fracture or defect. The molds were used many times (>20 cycles).

### Micro-CT imaging

The 3D dimensions of master microneedle array, microneedle array replica, and the PDMS mold were measured on the custom-build high resolution micro-CT facility at UNSW with voxel sizes of 1.36, 1.9, and 1.95 μm, respectively. The X-ray beam energy was set to 30–45 KeV and acquisitions took between 6 and 10 h with 65.7 GB projection data image collected by a high-quality 3072×3072 pixel flatbed detector (Varian 4343CB). Image reconstruction was carried out on the NCI supercomputing facility in Canberra utilizing 192 CPUs and 768 GB RAM via the 3D back projection based reconstruction algorithm^18^, producing about 16 GB of data per tomogram. The reconstructed 3D images were then filtered with an anisotropic diffusion filter for edge-preserving image de-noising^19^. A combination of watershed and active contour methods for segmentation of the gray-scale data were performed with the software called Mango as simple thresholding often does not produce correct surfaces as well as volumetric^20^. All visualizations were carried out using the open source software Drishti (version 2.6)^21^. Metrology was performed on the master microneedle array, the negative PDMS mold and Zeonor 1060R replica.

### Mechanical compression testing of thermoplastic microneedles

The mechanical strength of thermoplastic microneedles fabricated by soft embossing were measured and analyzed through a series of axial compression tests. Compression tests were performed on (1) single microneedles with a connecting reservoir, and (2) a microneedle patch consisting of 16 microneedles with connected reservoirs using a rheometer (Kinexus Rheometer, Malvern Instruments Ltd., Worcestershire, UK), the same instrument used to perform soft embossing. Throughout the experiment the lower platen of the rheometer was fixed and the upper platen approached microneedle tips with a range of velocities and displacements along the longitudinal axis of microneedles. Force and displacement during compression tests were continuously recorded so that force displacement curves could be plotted to determine the yield strength of microneedles. These were destructive tests so one sample was used per test.

For test (1) three single side-opened thermoplastic microneedles connected to a reservoir were tested. All the samples were fabricated by soft embossing using the same elastomeric mold. The microneedles had a 700 μm height, 150 μm tip length, and a reservoir depth of 180 μm. Single microneedles were attached by double sided tape to the lower platen of the rheometer. The upper platen of the rheometer was initially located 460 μm above the microneedle tip and programmed to approach the specimen with speeds of either 25, 35, or 45 μm s^−1^. The maximum displacement was fixed so that the upper platen stopped 300 μm above the lower platen. In test (2) the upper platen was initially 1.3 mm above the lower platen and traveled with a constant speed of 35 μm s^−1^ for 500 μm along the axial direction of a microneedle patch consisting of 16 microneedles with connected reservoirs. During compression, force and displacement of the upper platen were recorded for each sample.

### FEA of microneedle shaft design

The overall strength of an individual microneedle and its tendency to fail through buckling during skin penetration will depend on materials and overall shape in a way that can be modeled using FEA.

**Figure 1:**
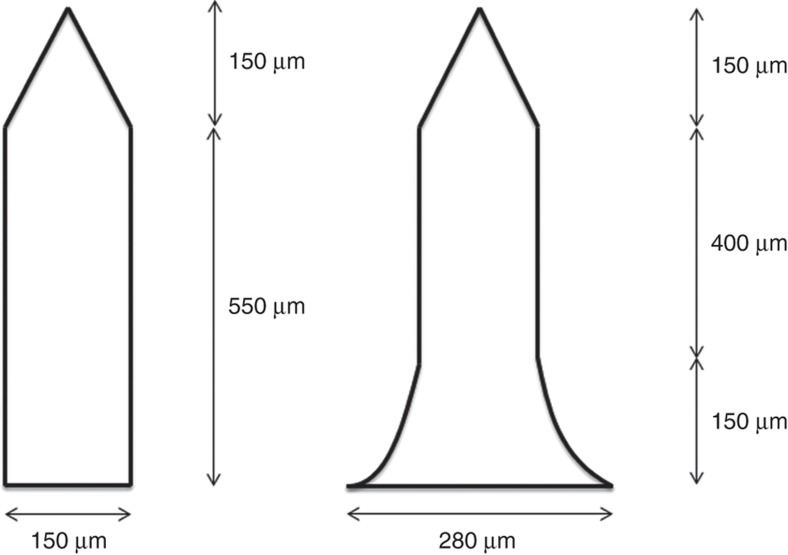
Microneedles geometries used for FEA.

Microneedle geometric design will affect the distribution of stress when the microneedle is loaded. The microneedle is more likely to fail at locations where stress is concentrated, specifically at sharp corners where the microneedle is joined to the back plane of the microneedle patch. Cracks that initiate microneedle failure will also propagate from regions of maximum tensile stress. Another feature of the microneedle design presented in this paper is a curved flange at the base of microneedle shafts to diffuse stress and strengthen the connection of the shaft to the microneedle back plane. The distribution of stress on loading was simulated by finite element analysis (COMSOL Multiphysics, COMSOL AB, Sweden, v4.3a) using the material properties of the thermoplastic. The material (Zeonor 1060R) was considered as a linear elastic material with Young’s modulus of 2100 MPa and Poisson ratio of 0.49. In this analysis the base connected to the microneedle was completely fixed where all other degrees of freedom of the microneedle were enabled. Maximum and minimum mesh element sizes were defined as 129 and 16.1 μm, respectively. Two microneedle designs were examined, a single open-channel microneedle with straight side walls and a single open channel microneedle with a flange at the base of its shaft, Figure 1.

### Oxygen plasma treatment

In order to facilitate filling of microneedle open channels and reservoirs by capillary pressure, the hydrophobic thermoplastic must be surface treated to reduce its contact angle to below 90°. Oxygen plasma treatment increases the free energy of the surface by creating hydrophilic, oxygen-containing groups such as carbonyl and carboxyl, esters on the surface^22,23^. Oxygen plasma treatment was performed on the thermoplastic microneedle arrays before biological experiments, using an oxygen plasma etcher (PE-250 Plasma etcher, Denton vacuum, USA) with 50 W RF power and 340 mTorr pressure for 20 min.

Contact angle measurements were executed on flat films of Zeonor 1060R based on the sessile-drop method by CAM 200 compact contact angle meter system (KSV Instruments Ltd., Helsinki, Finland) equipped with a C200-30 camera. Curve fitting software, KSV CAM Optical Contact Angle and Pendant Drop Surface Tension Software Version 3.95, was used to analyze collected images. All measurements were performed at room temperature with air as the light phase and water as the heavy phase. In order to assure the consistency and symmetry of the values, both right and left side angles of the droplet were analyzed. Deionized (DI) water 15 μL drops were placed on each sample by using a micropipette, and the image of each drop deposited on the surface was captured immediately.

### Skin penetration and drug delivery

The potential for microneedle skin penetration and drug delivery was tested “*in vivo*” using an euthanized rabbit ear. Studies were conducted using experimental procedures approved by the University of New South Wales Animal Care and Ethics Committee (ACEC) (Ethics Number 15/22A). Microneedle arrays consisting of 16 microneedles were introduced into the rabbit ear with an insertion velocity of 0.5 m s^−1^ using a commercial spring loaded applicator (Medtronic MiniMed Quick-Serter)^24^. The microneedle array was fixed to the middle of the applicator via a microscope coverslip using double sided tape. The microneedle array was dip-coated into a concentrated aqueous solution of fluorescein (sodium salt, F6377, Sigma-Aldrich Corp., St. Louis, MO, USA) at room temperature. The excess solution was carefully wicked away from the microneedle array using a tissue. Moderate pressure was applied for few seconds following microneedle patch insertion. The site of insertion was imaged following removal of the microneedle array using a Leica TCS SP5 STED confocal microscope (Leica Microsystems, Wetzlar, Germany). The penetration of fluorescein into skin was visualized by two-photon optical sectioning at various depths below the surface of the skin. A control experiment was required on the same tissue to quantify the rate of diffusion of fluorescein across skin without the microneedle array. A small drop of fluorescence solution was therefore placed on rabbit ear skin and imaged using the confocal microscope.

## Results

### Design and manufacture of single microneedles and microneedle arrays with reservoirs

**Figure 2:**
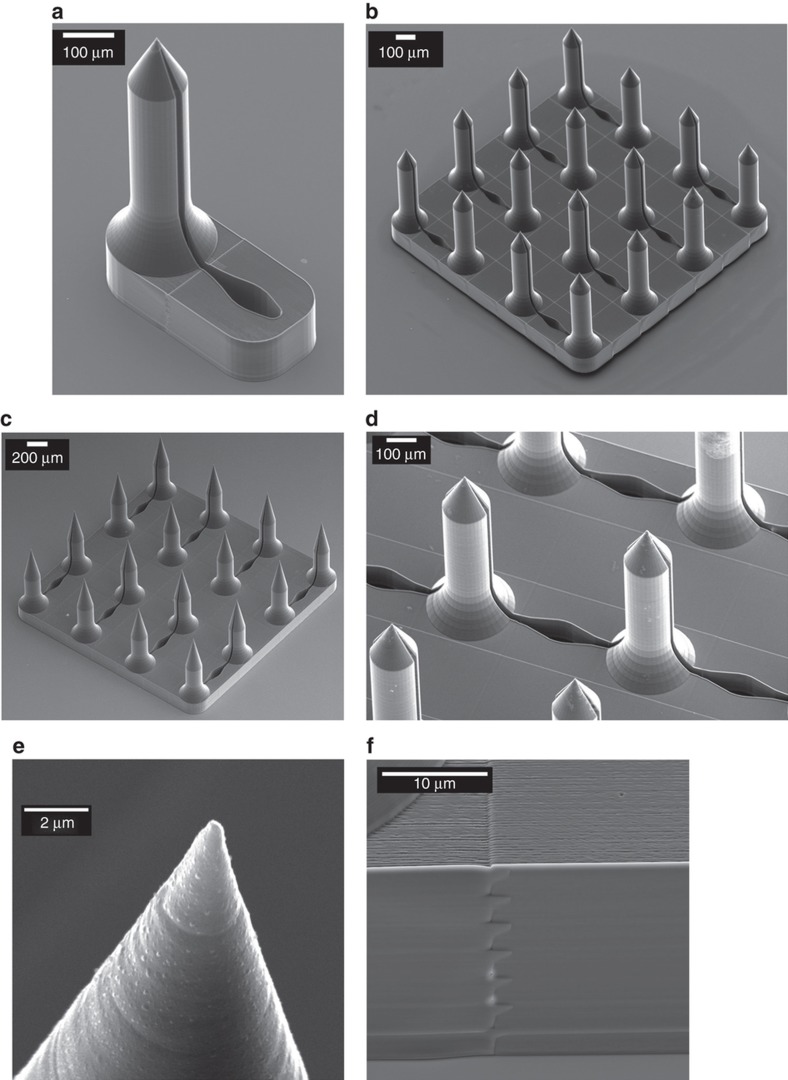
(**a**) SEM of fabricated single open-channel microneedle connected to a reservoir by 3D laser lithography. (**b**) A 16 microneedle array (2.17 mm×2.17 mm) with side channels connected to reservoirs fabricated by 3D laser lithography, microneedles have 700 μm total height, 150 μm tip height, 150 μm flange height. (**c**) A 16 microneedle array (2.17 mm×2.17 mm) with side channels connected to reservoirs fabricated by 3D laser lithography, microneedles have 700 μm total height, 350 μm tip height, 150 μm flange height. (**d**) Microneedles in the middle rows having two side-opened channels connected to different reservoirs. (**e**) Ultra-sharp microneedle tip. (**f**) Stitching of two adjacent blocks.

Open-channel master microneedles fabricated by two-photon polymerization (TPP) using the Photonic Professional GT system (Nanoscribe GmbH, Germany). Figure 2 shows a scanning electron micrograph (SEM) of a single microneedle with an open channel connected to a reservoir and a 16 microneedle and reservoir array. The microneedle open channel width gradually increases as it extends away from the shaft of the microneedle along the flange, and into the reservoir which is in the plane of the microneedle patch (Figures 2a–d). This design feature facilitates continuous drawing of fluid by capillary action into the reservoir until it has filled completely. The process of capillary filling with water tinted with food dye was videoed, and shown to occur in <200 ms (Supplementary Movie S1).

**Figure 3:**
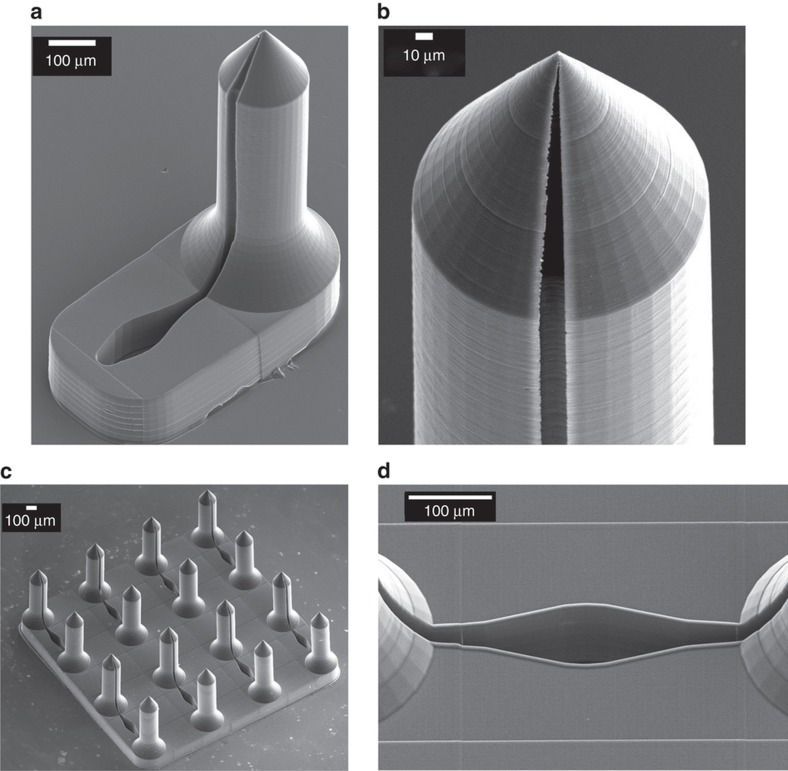
(**a**) Single side-opened microneedle replica with a fluid reservoir. (**b**) Precise replication of microneedle tip. (**c**) Microneedle array replica with straight projections. (**d**) Precise replication of features.

The write area of the Photonic Professional GT system using galvanic mirror scan control of the laser focal point was limited to a 250 μm×250 μm block, so *X*–*Y* microscope stage motion with *stitching* using Nanoscribe’s proprietary software Describe was required for printing 4×4 microneedle arrays (Figures 2b–d). Stitching leaves behind a small linear artifact which is placed so that it does not interfere with critical geometric features such as the needle tip (Figure 2e). Pairs (Figures 2b and c) or rows (Figure 2d) of microneedles were interconnected by channels and reservoirs. Each microneedle had a height of 700 μm with a 150 μm flange segment at its base. The taper angle of the microneedle tip was also varied (63.4°: Figures 2b and d; 77.9°: Figure 2c). Single microneedles and microneedle arrays were accurately replicated from TPP prototypes by soft embossing the medical grade thermoplastic Zeonor 1060R (Figure 3).

**Figure 4:**
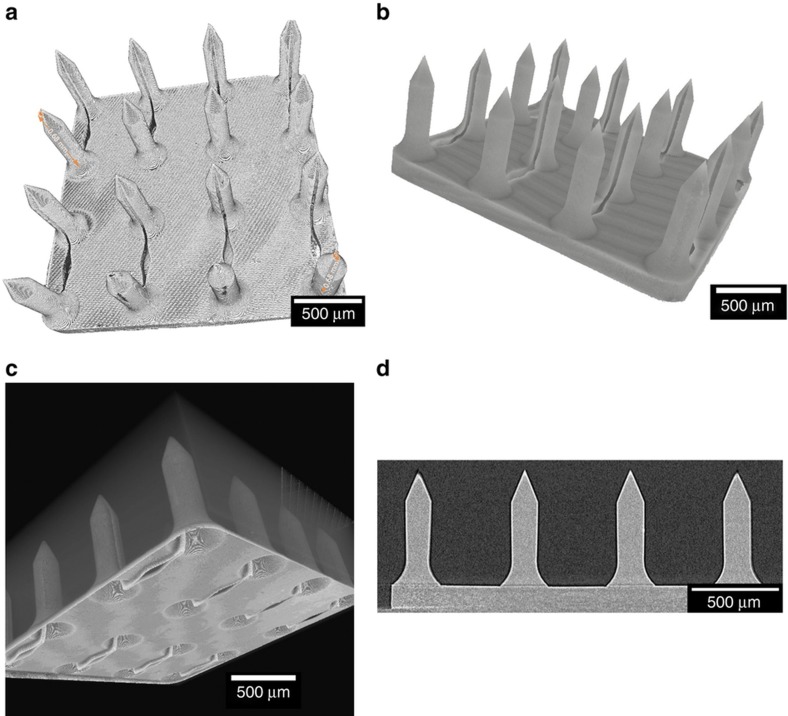
Micro-CT of (**a**) master microneedle array. (**b**) Microneedle array replica. (**c**) PDMS negative mold. (**d**) Cross sectional view of master microneedle array.

Microneedles replica, master and mold were scanned using 3D micro-CT to measure feature sizes (Figure 4). The imaging of master microneedle array verified that feature sizes were within 0.47% of the CAD drawing specification whereas microneedle array replica and PDMS mold were 3.48 and 3.44% smaller than the CAD drawing specification (Table 1).

### Bending force FEA of microneedles

**Figure 5:**
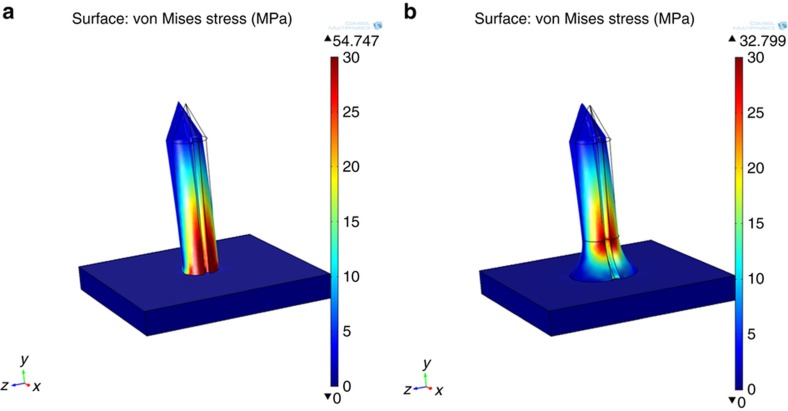
COMSOL FEM simulation of microneedle bending and stress concentration when a 20 mN lateral load (along *x-*direction) is applied to the needle tip taper region. Color map shows surface stress (**a**) no flange at base of the microneedle. (**b**) Flange at base of the microneedle.

Microneedles are most likely to fail where stress is concentrated. Figure 5 shows a COMSOL (COMSOL Multiphysics, COMSOL AB, Stockholm, Sweden) FEM simulation of microneedle bending and stress concentration when the microstructures experience a static lateral load of 20 mN applied at the microneedle tip. Microneedles are most likely to fail at the transition between the body of the microneedle and the support member where the maximum von Mises stress was 54 MPa (Figure 5a). A flange with radius of 140 μm was introduced to the base of the microneedle to minimize stress concentration. For the flanged design (Figure 5b) the maximum von Mises stress (34 MPa) occurred above the flange and not at the transitions between the base and the microneedle body. The values of von Mises stress obtained for the flanged design was less than the yield strength of Zeonor 1060R (53 MPa)^25^.

### Mechanical compression testing of microneedles

**Figure 6:**
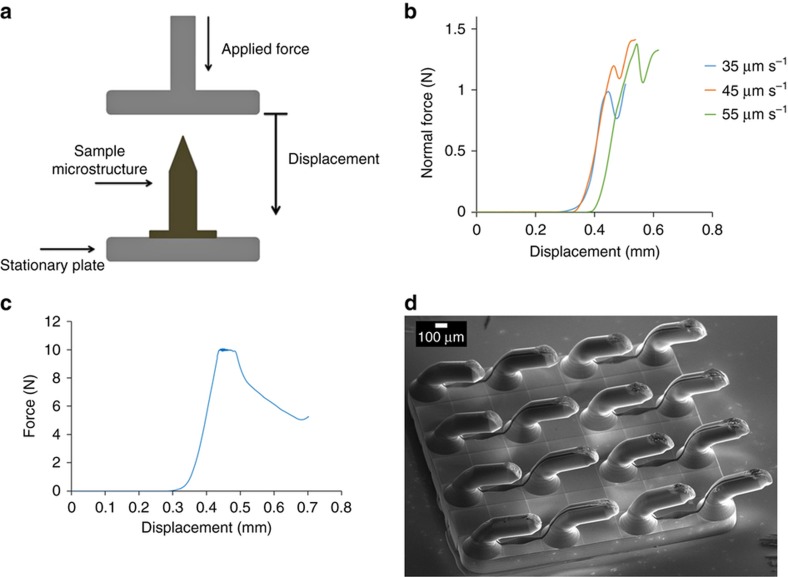
(**a**) Schematic setup of compression test on microneedles performed by rheometer. (**b**) Force (N) versus displacement (mm) curves of compression tests with speeds of 35, 45, and 55 μm s^−1^, on three separate microneedles connected to a reservoir. (**c**) Force (N) versus displacement (mm) graph of a compression test on an array of 16 microneedles by applying 35 μm s^−1^ speed. (**d**) SEM image of the microneedle array with tip heights of 150 μm after compression test.

Dynamic loading tests were conducted to determine the yield strength of microneedles when force was applied along the long axis of microneedles. Three geometrically identical microneedles (Figure 3a) were tested at different velocities (35, 45 and 55 μm s^−1^). As can be seen from Figure 6b, force increases upon the first contact of the rheometer’s upper platen reaching a maxima, followed by a minima and a secondary maxima. The first maxima on each curve corresponds to the failure load that resulted in permanent deformation of the structures. The subsequent force increase corresponds to compression of the microneedle base. The lag time between the start of recording varies because different distances are traversed before the upper platen makes contact with the microneedle tip. The slope of the initial force displacement curves was identical; however, velocity was directly related to the failure load of the specimen.

Figure 6c shows the force versus displacement curve for a microneedle patch array consisting of a 4×4 array of 700 μm microneedles and a tip taper angle of 63.4°. The displacement was linearly related to the applied force up to failure at 10 N (Figure 6c). During compressive failure the force was approximately constant over a 100 μm displacement range. A SEM of the microneedle patch following the axial compression test (Figure 6d) showed that microneedles where permanently deformed by bending and compression, without obvious fragmentation.

### Delivery of fluorescein into skin

**Figure 7:**
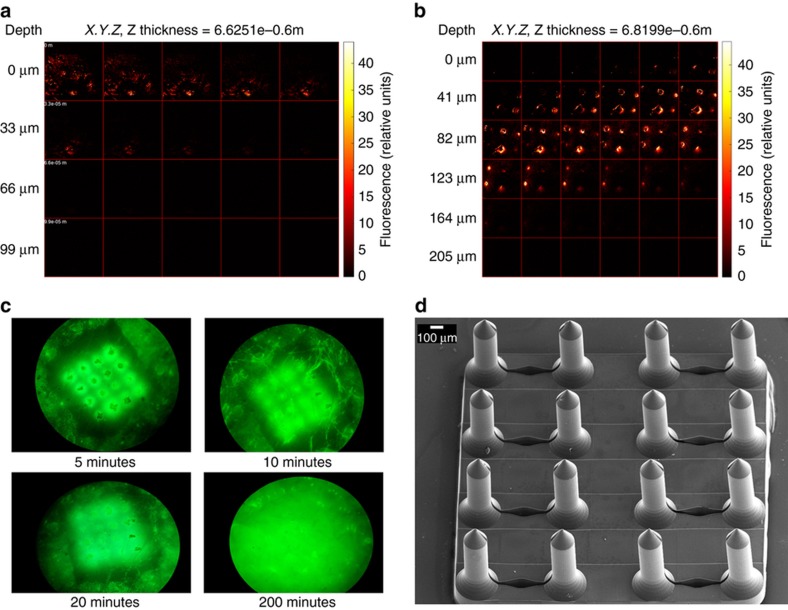
Multiphoton microscopy image of (**a**) topical application of fluorescein solution on tissue surface. (**b**) Fluorescein solution diffusion underneath the skin surface of rabbit ear. (**c**) Confocal microscopy image showing the diffusion of fluorescein solution underneath the skin surface of rabbit ear over four time intervals. (**d**) SEM image of the array of microneedles after removal from rabbit ear.

**Figure 8:**
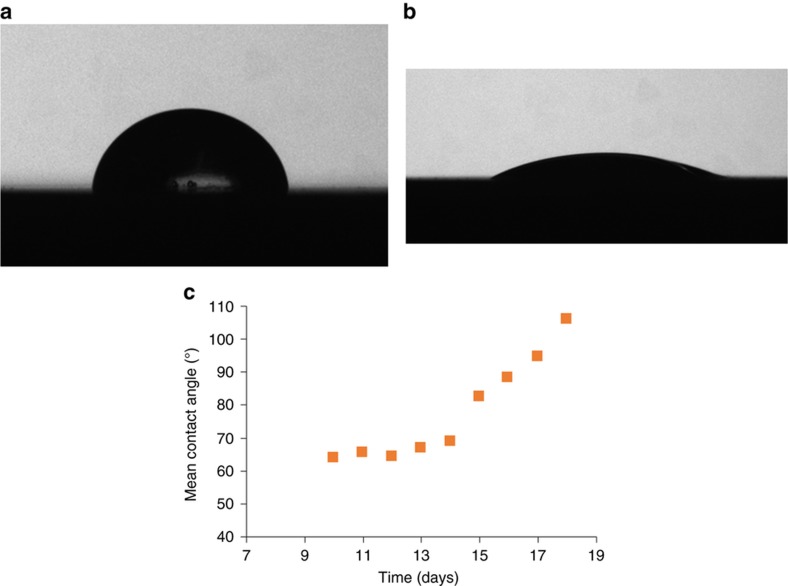
A 15 μL of DI water droplet on Zeonor 1060R film (**a**) with no surface modification. (**b**) With surface modification measured at day 1. (**c**) Contact angle measurement for evaluation of hydrophobicity recovery 10–18 days after surface modification.

Multiphoton confocal microscopy was used to measure the depth of penetration of fluorescein solution delivered by a 4×4 microneedle array into a cadaveric rabbit ear. The *Z* image stack is shown in Figures 7a and 8b. Figure 7a shows a control experiment where a drop of solution applied to the rabbit skin without application of a microneedle patch. The fluorescein signal disappeared at 66 μm below the skin surface. Figure 7b shows microneedle patch insertion points with tracking of fluorescein to a depth of at least 160 μm. Figure 7c shows the penetration of fluorescein into the skin over 3 h. Figure 7d shows the SEM image of microneedle patch taken after insertion and removal from the rabbit ear. As can be seen from the Figure 7d, no fracture or bending of microneedles was observed, microneedle array was washed and sputter coated with gold coating prior to SEM imaging.

## Discussion

The methods in this study would allow production of complex microstructures. The microneedle geometries generated by TPP would be extremely difficult to achieve through subtractive manufacturing methods such as deep reactive ion etching (DRIE), laser machining or chemical wet etching. Thus, microneedle designs are not restricted by the physics of machining and etching, but based upon functional and structural criteria. Doraiswamy *et al.*^26^ fabricated microneedles by TPP from Ormocer hybrid materials with 750 μm height and 200 μm base diameters. Ovsianikov *et al.*^27^ also fabricated 800 μm tall microneedles by TPP with base diameters ranging from 150–300 μm. Organically modified ceramic-Ormocer US-S4 has been used for manufacturing arrays of out-of-plane and in-plane hollow microneedles.

Manufacture times were reduced to less than 20 min per patch by thermoplastic (Zeonor 1060R) replication of TPP prototypes using soft elastomeric molds. The embossing process was automated with Peltier heating above the glass transition temperature and cooling below ambient. Zeonor’s low melt viscosity at 60 °C above its glass transition temperature facilitates mold penetration without the need to apply high pressure. The elastomeric molds remained undamaged after at least 22 replication cycles, due to the use of very low embossing forces (~19 N). During demolding the silicone rubber deforms with low force, and does not stress or fracture the fragile microstructure. While it was not possible to replicate microneedles with a lumen (Supplementary Figure S1), because elastomeric cores do not have structural stability, and detach during demolding, it was possible to replicate open-channel microneedles with moderate undercut angles and high aspect ratio reservoirs. open-channel designs appear to perform just as well as hollow microneedles for rapid filling by capillary action (Supplementary Movie S1) or transfer of fluorescein across the epidermis. The mechanical properties of PDMS do not degrade over several months^28^. Future work will examine the feasibility of scaling soft embossing of TPP prototypes by injection molding or reel to reel embossing.

Microneedles may experience different forces such as buckling, bending, axial, lateral, and skin resistive forces during penetration into the skin. Bending of microneedles may occur as a result of inclined insertion of the microneedle patch into the skin, or alternatively uneven skin surface can induce bending on the microneedles. For microneedles presented in this paper bending force can additionally be applied during fabrication processes. There is a high chance of microneedle failure by bending force applied when separating the microneedle master prototype from PDMS elastomeric mold after curing and when demolding soft embossed microneedles from the PDMS negative mold.

The maximum stress due to bending takes place at the microneedles base at the time of skin penetration. The microneedle bending force can be determined by Euler–Bernoulli beam theory. The maximum lateral (bending) force without causing breakage in the microneedle is defined by:

(1)FBending=σyILr

where, *σ*_*y*_ (N m^−2^) is the yield strength of the material, *r*(m) is radius of the microneedles, (m^4^) is the area moment of inertia, (m) is the length of the microneedle, and (*N*) is the lateral force on the microneedle tip. The yield strength of Zeonor 1060R is 53 MPa (Ref. 25). Therefore, according to Equation (1), a single microneedle, with geometries presented in Figure 1, without a channel or flange, would yield at bending force of 25 mN. These values are consistent with the maximum stresses obtained from FEA results. FEA shows that the flange design distributes the surface stress when a 20 mN load was applied to the side of the needle tip in the taper region (Figure 5). The simulation results show that without a flange the microneedle will break at its base since the maximum von Mises stress was 54 MPa. For the flange design the maximum von Mises stress of about 34 MPa was occurring just above the flange and not at the transitions between the base and the microneedle body.

In addition, microneedles will fail by buckling if the shaft diameter is too small for the applied force. The critical load buckling force *F*_Buckling_ for bucking is calculated by Euler’s formula:

(2)FBuckling=π2EI(KL)2

Here, *E *(Pa) is the modulus of elasticity of the material, *I*(m^4^) is the area moment of inertia, *L* (m) is the shaft length, and *K* as constant called the effective length factor which depends on the mode of column support. In this paper all the fabricated microneedle shafts are fixed at one end to a patch base, whilst the tip is free to move laterally (*K*=2). Therefore, according to Equation (2) for a Zeonor 1060R microneedle with Young’s modulus of 2100 MPa, 700 μm total height that includes a 150 μm flange and 150 μm tip the failure buckling force would be ~0.4 N.

A needle tip will penetrate the human epidermis if it exerts tensile stress at the point of contact beyond the ultimate strength of skin which is about 27.2±9.3 MPa. The ultimate strength of skin varies according to age and body location^29^. The sharper the needle tip, the more concentrated the tensile force at the point of contact. Microneedle tip sizes smaller than the stratum corneum cells (corneocytes) sizes (30 μm) can penetrate the skin more easily than larger tip sizes. This is due to the fact that in small tip sizes the penetration force rather than being applied to a large area of the tissue will be only applied on the individual cells or in between the cells^7^.

Khanna *et al.*^30^ studied the effect of the tip sharpness of hollow microneedles on human cadaver skin penetration force. Insertion forces were inversely related to tip area, and decreased markedly from 4.75 N for a blunt tip (tip area 14 400 μm^2^) to 0.1  N for the sharpest microneedle (tip area: 186 μm^2^). Davis *et al.*^31^ experimentally measured the forces required for insertion of microneedles into human skin based on microneedles tip diameters. In this study, it was concluded that for microneedles with interfacial tip areas less than 5000 μm^2^ the insertion force required to penetrate human skin is less than 0.4 N (Ref. 31). Wang *et al.*^32^ reported the required insertion force of a sharp beveled tip microneedle to be 0.275 N for insertion into excised porcine skin. This study confirms that the tip geometry and sharpness are significant factors in reducing the required force of microneedle insertion^32^. The microneedles fabricated in our study through a soft embossing process had tip diameters much smaller than 1 μm, and thus had tip areas of less than 0.75 μm^2^. Therefore, the insertion force for microneedles should be considerably smaller than 0.4 N. The minimum failure load (1.04 N) observed in Figure 6b for 35 μm s^−1^ speed was much higher than the required insertion force reported in the human skin studies^30,31^. In addition, the rabbit ear insertion experiment demonstrated that the microneedle patch was mechanically robust enough to remain intact after penetration of rabbit ear (Figure 7d).

Due to the hydrophobic nature of cyclic olefin polymer Zeonor 1060R, oxygen plasma treatment was required to make the surface hydrophilic so that the microneedle could generate surface tension for passive filling of the open channel and reservoir. We observed passive filling of microneedles and reservoirs that were treated by oxygen plasma in less than 200 ms, while untreated hydrophobic devices did not fill with water. The measured contact angle of untreated Zeonor 1060R was 88.25° (Figure 8a) while the contact angle of DI Water on the surfaces of a sample after surface modification on days 1 was 21.57° (Figure 8b). In order to further explore the restoration of Zeonor 1060R hydrophobicity, contact angle measurements were performed on a sample for 9 consecutive days; 10 days following surface treatment, showing almost total restoration of the initial hydrophobicity of Zeonor 1060R within about 2 weeks (Figure 8c).

At microscale, the forces that determine filling of a capillary are surface tension and viscous drag. Gravity can be neglected because the specific gravity of fluid is negligible compared to surface tension (Bond number<1). Flow along a microfluidic open channel is viscous because Re<1. The flow rate is approximated by equating the viscous pressure drop along a cylinder (Hagen-Poiseuille equation: 8 *μLv*/*r*^2^) with the surface tension drawing fluid into the hydrophilic cylinder (Young-Laplace equation: 2*γ* cos*θ*/*r*). The velocity of the meniscus during filling of a capillary depends on *L*, the length of capillary filled with fluid:

$$v(L)=\frac{r\gamma \cos\theta }{4µL}$$

where *r* is the capillary internal radius, *θ* the contact angle, and *γ* the surface tension. The capillary filling time, *T*_fill_, is found by integrating with respect to channel length:

$$T_{fill}=\int_{0}^{L}\frac{\text{d}x}{v(x)}=\frac{2µL^{2}}{r\gamma \cos\theta }$$

For example, a 700 μm capillary with radius 15 μm would fill with blood (*γ*=55×10^−3^ N m^−1^; *μ*=4×10^−3^ Pa s, *θ*=70°) in 14 ms (Re=0.1, Bond number=0.09). For open-channel flow the filling time would be similar to this value because viscous pressure drop and capillary pressure both scale approximately linearly with respect to wetted perimeter.

Once the open channel has filled it may leak if the fluid pressure exceeds a leakage pressure (*P*_leak_). Geometric considerations lead to a formula, which relates leakage pressure directly to contact angle, surface tension and inversely to channel width *w*:

(3)Pleak=2γsinθw

Microneedle side channels were designed with a 20-μm wide opening so that the calculated leakage pressure was 5.2 kPa (*γ*=55×10^−3^ N m^−1^, *θ*=70°) which is higher than blood capillary pressure (2.0–4.7 kPa)^33^. Thus, open channels are more likely to leak if their contact angle is less than or the channel opening is >20 μm.

## Conclusion

A reliable and relatively uncomplicated method for manufacturing novel microneedle arrays integrated with open-channel microfluidics for subcutaneous fluid sampling and drug delivery was developed. Negative elastomeric molds were used to replicate TPP prototypes by hot embossing the cyclic olefin polymer Zeonor 1060R. It was possible to produce multiple polymeric replicas with almost identical geometry to the master and CAD design using the same negative elastomeric mold. Microneedle designs with open microchannels connecting microneedle flow to a reservoir were easily replicated by soft embossing. Hydrophilic open-channel microneedles fill rapidly by capillary tension, and can deliver fluorescein tracer into skin of rabbit ear.

Moreover, the deflection of microneedles and stress distribution under a lateral (bending) force was investigated by FEA using COMSOL Multiphysics software. Another novel design feature, the flanged base, was added to the design to reduce stress concentration and fracture at the base of microneedles.

In conclusion, the use of 3D laser stereolithography to create master prototypes, with replication by soft-embossing is a significant advance in the field of microneedle manufacture. Microneedle design can be based primarily upon structural and functional modeling. Novel geometric features such microneedle open channels connected to microfluidic reservoirs that are directly rendered from CAD drawings go way beyond what is possible by subtractive fabrication methods. Replica molding of thermoplastic microneedles has the advantage of low material input and capital equipment costs (versus DRIE for silicon) with potential for large-scale manufacture of novel microneedle designs. Future advances will focus on application of lab-on-a-chip to point-of-care healthcare delivery.

## Figures and Tables

**Table 1:** 3D micro-CT measurement on a 16 microneedle array master, replica, and PDMS mold

	Master	PDMS mold	Zeonor replica
Height (mean±standard deviation)	696±11.6 μm	675±3.83 μm	675±4.82 μm
Percentage of change (shrinkage) from CAD drawing	0.47%	3.44%	3.48%
Micro-CT voxel size	1.36 μm^3^	1.95 μm^3^	1.9 μm^3^
