# Supplementary material for: High-fidelity replication of thermoplastic microneedles with open microfluidic channels
Source: Microsyst Nanoeng. 2017 Oct 9;3:17034. doi: 10.1038/micronano.2017.34 (PMC6445010; doi:10.1038/micronano.2017.34)
Supplement: Supplementary Information [file micronano201734-s1.pdf]

Supplementary file

High fidelity replication of thermoplastic microneedles with open microfluidic channels

Zahra Faraji Rad[1,2,3], Robert E. Nordon[1], Carl J. Anthony[2], Lynne Bilston[4], Philip D. Prewett[2], Ji-Youn Arns[5], Christoph H. Arns[5], Liangchi Zhang[3] and Graham J. Davies[1]

Microsystems & Nanoengineering (2017) **3,** 17034; doi:10.1038/micronano.2017.34; Published online: 9 October 2017

Parameters such as laser power, scan speed of the laser, hatching between layers, stitching, and slicing distance play a key role in process optimisation. In addition to the aforementioned parameters, the microscope objective characteristics and the photosensitive material have significant influence on the final structure. The key aims of the fabrication parameters optimisation process were to define the best mechanical stability and resolution of the microneedles structures with the shortest possible writing time. Several optimisation runs were conducted to determine the ideal configuration of parameters. In this section a few examples illustrating the optimisation process are presented.

Scan speed defines the rate of laser beam movement through the liquid material in order to polymerise the photoresist material. High scan speed reduces the writing time due to faster movement of the laser beam; however, this could form structures which are not mechanically stable that shrink with more surface roughness due to a suboptimal dose during polymerisation. Scan speed only contributes to polymerisation in *XY* direction, and not in *Z* direction. Supplementary Figure S2 shows a microneedle fabricated with high scan speed of 100 000 $\mu m\ s^{-1}$ and laser power of 80 mW out of IP-Dip photoresist with a 63× magnification objective. For this structure a minimum slicing distance of 0.5 µm and maximum of 1.2 µm were chosen which were relatively large. Adaptive slicing feature was chosen for fabrication of microneedles. During slicing the object the adaptive slicing applies the slope at the current layer to define where to slice the next layer. A light slope results in a smaller slicing distance where a steep slope results in a higher slicing distance. The final slicing distance will be in the range of minimum and maximum slicing distance determined. The combination of a high scan speed and large distance between layers leads to the creation of structures which are not mechanically stable.

A combination of 27 microneedles was fabricated to find the ideal recipe for fabrication of microneedle arrays using IP-S photoresist and the 25× magnifications microscope objective. This objective has NA of 0.8 and working distance of 390 µm where 63× magnifications microscope objective has NA of 1.4 and working distance of 190 µm. Due to larger working distance of 25× magnifications microscope objective and the larger voxels caused by the smaller NA the writing speed subsequently increase by using this microscope objective. As a result it is possible to expand the volume and write high and large structure. Laser power was kept constant at 100 mW while changing the scan speeds from 60 000–100 000 $\mu m\ s^{-1}$ with 20 000 $\mu m\ s^{-1}$

a b

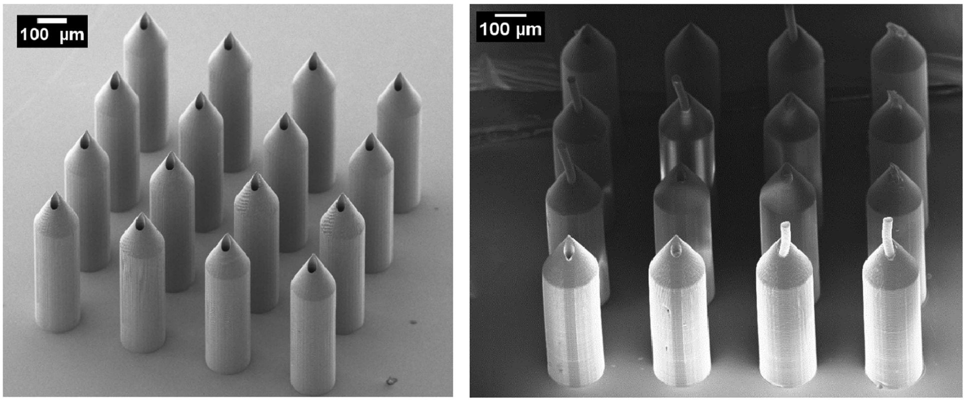

Figure S1 (**a**) Master microneedles with 600 µm heights, base diameters of 150 and 250 µm spacing between microneedles. The internal channels have diameters of 30 µm. (**b**) Thermoplastic hollow microneedle patch manufactured through soft embossing process.

[1]Graduate School of Biomedical Engineering, University of New South Wales, Sydney, NSW 2052, Australia; [2]School of Mechanical Engineering, University of Birmingham, Birmingham B15 2TT, UK; [3]School of Mechanical and Manufacturing Engineering, University of New South Wales, Sydney, NSW 2052, Australia; [4]Prince of Wales Clinical School, University of New South Wales, Sydney, NSW 2052, Australia and [5]School of Petroleum Engineering, University of New South Wales, Sydney, NSW 2052, Australia
Correspondence: Zahra Faraji Rad, (E-mail: zahra.farajirad@uq.edu.au)

Q2

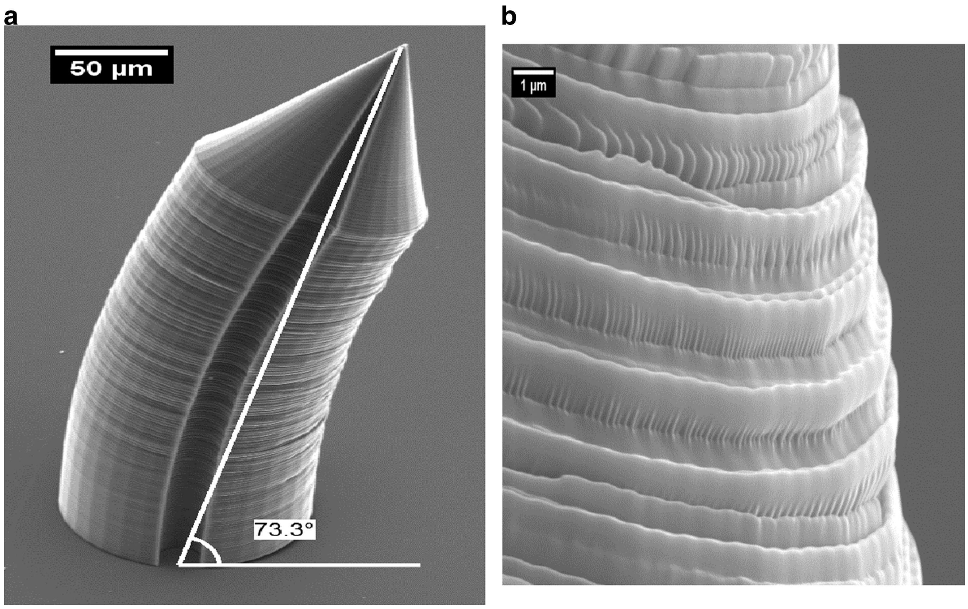

Figure S2 (**a**) Microneedle bent over due to incomplete polymerisation of the photoresist, (**b**) visible layers pattern on the microneedle profile.

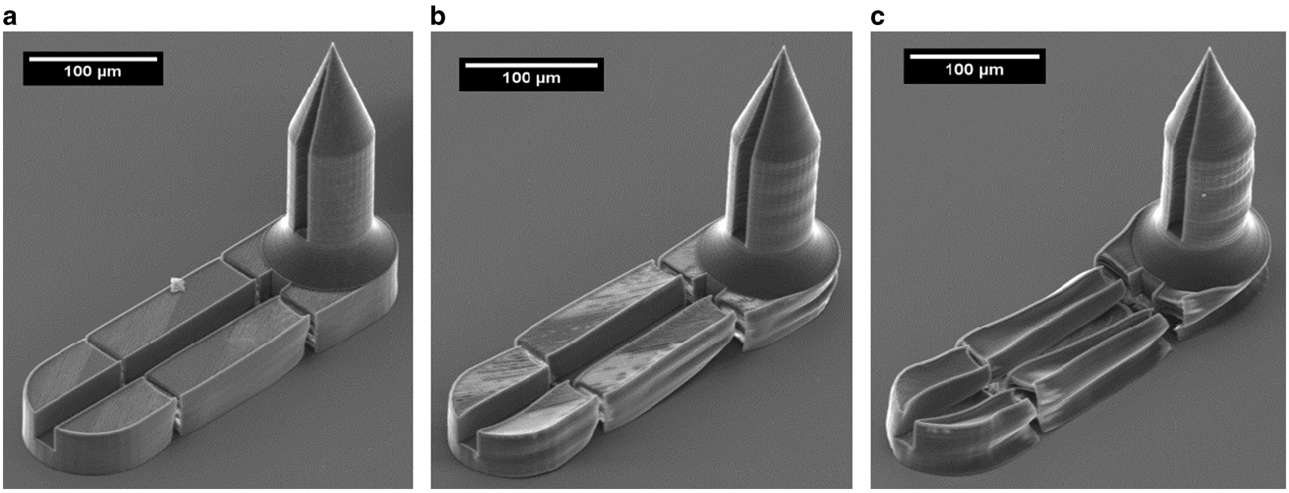

Figure S3 Comparison of maximum slicing distance of (**a**) 0.5 µm, (**b**) 1 µm, and (**c**) 1.5 µm for 60 000 µm s^{-1} scan speed and hatching every 7 layers.

increments and hatching every 3, 5, and 7 layers while changing the maximum slicing distance to 0.5, 1, and 1.5 µm. Figure S3 exhibits the influence of slicing distance on the final blocks and their stitching. Figure S4 illustrates the effect of hatching between layers. The hatching is used for filling the interior of the layers and also to compensate or reduce the accumulation of internal stress occurring from polymerisation shrinkage. Tight hatching patterns results in denser polymerisation of the structure, but significantly increase the total writing time. Finally, a combination of 100 mW laser power, 60 000 µm s^{-1} scan speed, 0.3 and 0.5 µm minimum and maximum slicing, and hatching every seven layers were selected as the optimal parameters for the abovementioned objective and the photosensitive material.

Keeping the slicing distance and scan speed to minimum will lead to complete polymerisation of photo-curable materials and superior resolution, however, these settings will dramatically increase the writing time for each structure. Therefore, it is important to optimise writing parameters so that fabrication of structures is mechanically stable and have optimal geometric resolution. Exposure to UV light for 20 min after laser printing and development was added to completely crosslink the photoresist. Figure S5 shows an example of a structure which has not been fully polymerised, some of rectangular blocks were peeled off from the structure and large bubbles were formed on the surface after de-vacuuming the sample for SEM imaging.

Figure S6 show how high laser power has over-exposed the layers and formed non-homogeneity on the structure.

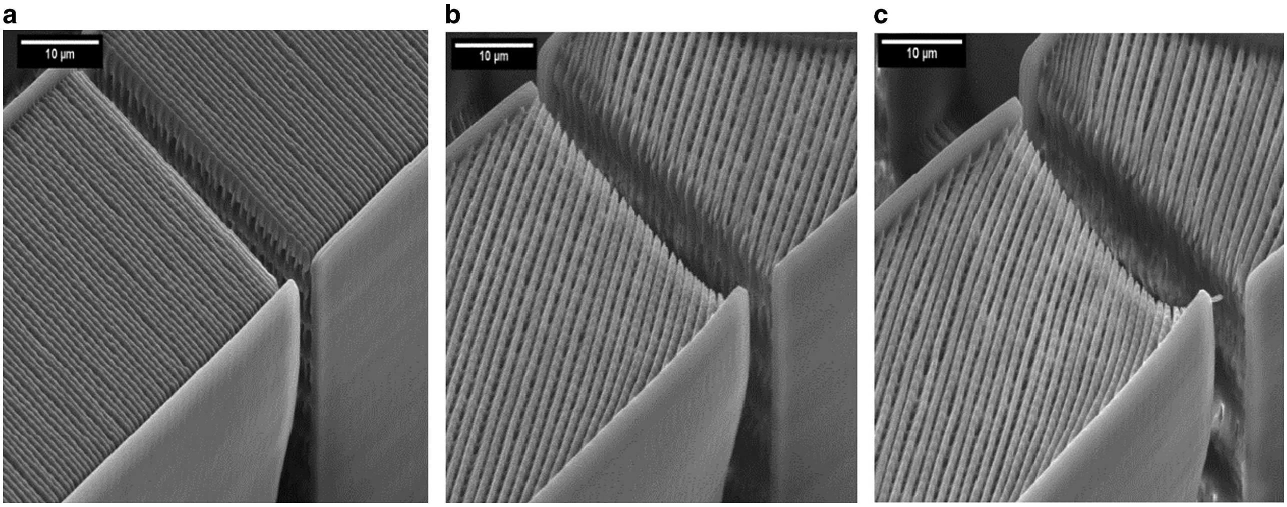

Figure S4 Comparison of hatching every (**a**) 3, (**b**) 5, and (**c**) 7 layers for 60 000 μm s^{-1} scan speed and maximum slicing distance of 0.5 μm.

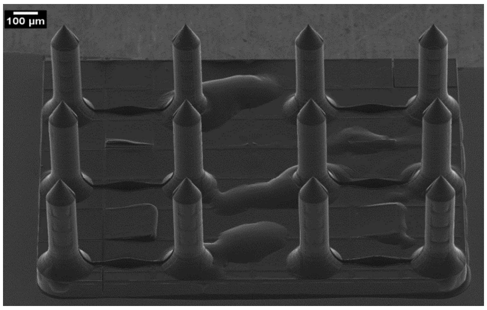

Figure S5 Formation of bubbles on microneedle patch due to incomplete crosslinking of support membrane photoresist.

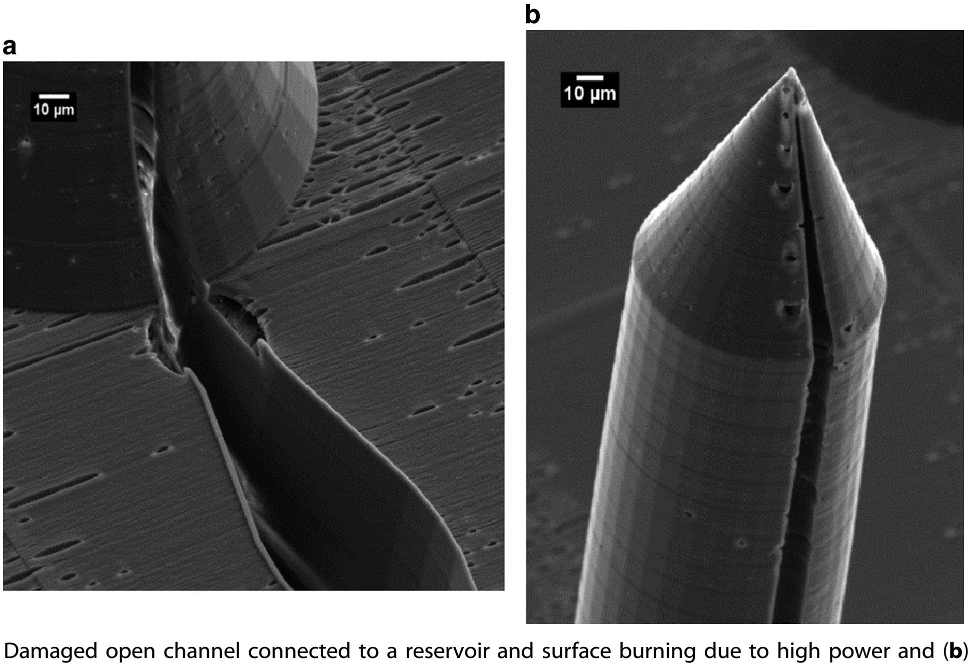

Figure S6 (**a**) Damaged open channel connected to a reservoir and surface burning due to high power and (**b**) tip burning.
